# Measuring the decent work of knowledge workers: Constructing and validating a new scale

**DOI:** 10.1016/j.heliyon.2023.e17945

**Published:** 2023-07-07

**Authors:** Yan Yan, Yuqing Geng, Juan Gao

**Affiliations:** School of Business, Shanghai Dianji University, Shanghai, China

**Keywords:** Decent work perception, Knowledge workers, Scale development, Grounded theory, Quantitative analysis

## Abstract

Through two and half decades, decent work (DW) has been studied mainly on unemployment and underemployment to promote workers' well-being, overlooking the importance of understanding knowledge of workers' (KW's) well-being in the workplace. However, the conflict between organizational performance and KW's well-being has intensified with technology development and the digital economy. This study constructs and validates a new scale for measuring the decent work perception (DWP) of KW in the context of knowledge management (KM). First, 27 in-depth interviews are conducted based on previous research results and grounded theory, resulting 4 dimensions consisting of 32 initial items identified by the KWs as reflecting their perception of DW. The 4dimensions of DWP are work security, respect & support, self-value, and professional skills. Second, 212 KWs participate in the preliminary survey, identifying the initial questionnaire's validity and selecting 13 items from the original questionnaire. Finally, 554 questionnaires are collected for the formal survey. According to exploratory factor analysis (EFA) and confirmative factor analysis (CFA), the 13-item decent work perception scale (DWPS) has reasonable reliability and validity. Considering the characteristics of work challenges, work autonomy, and career commitment of KWs, this study contributes to the knowledge of respect & support, self-value, and professional skills on DWPS. It is concluded that the DWPS can be applied to measure DW for KWs. As an effective measurement tool for KWs, this scale is also crucial for helping employees achieve their career goals, and managers understand KWs' demands theoretically and practically.

## 1 Introduction

As a way of maintaining social sustainability, work is a vital link between workers, organizations, and society [1]. With technology and the digital economy, enterprises are becoming more competitive. Although economic development has brought job opportunities and increased incomes, the tension between employee wellbeing and organizational performance achievement has come to the fore [2]. The employee overload phenomenon has emerged frequently in certain companies and positions [[3], [4], [5]]. For instance, according to China Moderate Labor Research Center, knowledge workers with higher education work longer than less-educated workers [6]. For young and well-educated workers, private companies force them to work voluntary overtime under the pretext of self-fulfillment and continuous improvement [7]. Following COVID-19, working from home has become more prevalent, resulting in longer working hours. A literature review of COVID-19's impact on working from home indicates that overworking is one of the main challenges remote workers faces [8].

As work forms change, DW is challenged [9]. In addition to creating psychological pressure on employees, overwork negatively affects work performance, organizational innovation, and sustainable development. Overwork can increase the likelihood of burnout, psychological detachment [10], and even suicide attempts among employees [2]. Additionally, heavy work on time and effort investment negatively impacts individual work performance and increases turnover intentions [11]. Further, overworked KWs have a detrimental effect on organizations. Educated KWs contribute significantly to organizational innovation and are valuable resources for organizations seeking a sustainable competitive advantage [12]. As a result of the increased mobility of such employees, enterprises risk losing organizational knowledge, thereby reducing their ability to build a competitive advantage [13]. Even though overwork negatively affects psychological pressure and work performance, why are KWs still willing to work overtime? That is because KWs create value for themselves through knowledge creation. In addition, they have a strong commitment to their careers. Voluntary overworking negatively affects social development sustainability and should not be a tool to deprive KWs of their well-being [14].

DW, a way of evaluating workers' well-being, is associated with workers' physical health directly and indirectly, workers' creativity and innovation ability, as well as its significance for the company's competitive advantage [15,16]. Developing DW for KWs is urgent in human resource management practice. Because with the advent of a knowledge-based economy, DW for KWs can improve individual creativity and innovative behavior, organizational innovation performance, and sustainable competitive advantage.

Scholars have paid attention to DW since the 1990s. DW was first proposed to the International Labor Organization (ILO) by Juan Somavia in 1999. Researchers have extensively researched the definition, measurement, and improvement of DW in unemployment and underemployment over the past two decades. However, there are some limitations. (1) Lack of theoretical foundations for definition and measurement from an individual perspective. Research on DW from an individual perception perspective is still exploratory. Research results and theories can be referred to in a limited number of cases. Scholars have not yet reached a consensus on the DW meaning from an individual level. They have not developed a solid theoretical basis for measuring DW individually. (2) Lack of DWPS considering KW characteristics. Existing DW scales focus on the basic functions of DW: satisfying the underlying needs of workers [17]. KWs have specific features of knowledge creation and career commitment [18,19], but previous measurement research on DW does not reflect KWs’ high-level needs.

Although scholars have conducted extended studies on the concept and scale development of DW for manual workers, they need to pay more attention to understanding knowledge workers, such as overwork and burnout. Evaluating knowledge workers' decent work status has become a crucial research topic in the theoretical field. Moreover, human resource management (HRM) is eager for support at the individual perception of DW to facilitate the company's understanding of KWs' physical and psychological status to lay the foundation for bettering working conditions. Measuring DW for KWs needs extensive attention in theoretical and practical fields. This study illustrates the research purposes below based on the arguments developed above.

This study was conducted as follows. (1) We developed the structure of DWP based on a literature review and in-depth interviews with 27 participants. (2) We conducted a preliminary survey by ground theory to develop the initial questionnaire of the DWPS and revised the items based on EFA and CFA. (3) We carried out a formal survey and tested the reliability and validity of DWPS.

By developing DWPS, the significance of this study is shown as follows. (1) DWP can help managers motivate knowledge workers based on their needs. The four-dimensional structure of DWPS, consisting of job security, respect & support, self-value, and professional skills, reflects knowledge workers' innovation behavior and career commitment. (2) DWPS can assist organizations in understanding the perceived status of decent work among knowledge workers, including psychological, environmental, and social aspects. It can also formulate HRM practices that will enhance knowledge workers’ well-being.

This paper is structured as shown below. “Literature review” reviews prior studies on DW. Grounded theory, known as a method of quantitative analysis, is applied to develop initial scale of DWPS in the section “Initial scale construction based on grounded theory.” Section “Quantitative method” develops the scale and evaluates its reliability and validity through preliminary survey and formal survey. In the “Conclusion and discussion” section, research results are concluded, and practical and theoretical implications are discussed. We discuss the limitations and future research direction in this study are shown in “Limitations and future research directions.”

## 2 Literature review

### 2.1 Concept development

#### 2.1.1 Definition at the macro level

DW was initially proposed by Juan Somavia, ILO director, in 1999. The ILO's Decent Work initiative aims to help all workers achieve free, equal, secure, and dignified work (ILO, 1999). The ILO's DW emphasizes the need to create a sustainable and fair employment environment for workers, to continuously promote social protection for workers, and to continuously strengthen social dialogue for workers. Although the ILO has clearly defined the scope and goals of DW, scholars argue that more is needed. The definition of ILO emphasizes objective economic indicators of DW, such as unemployment rates and salaries, neglecting subjective perceptions of working conditions [20]. Therefore, it is necessary to define DW from an individual perspective in future research.

#### 2.1.2 Definition at the micro level

The United Nations introduced the concept the Sustainable Development Goals (SGDs) in 2019, emphasizing the importance of good employment conditions and social protection. The SDGs is aiming at fostering sustainable and inclusive economic development, employment, and decent working conditions. This goal entails transforming research focus on macro-level DW to the micro level of DW, which means the focus shifts from countries' status quo to workers’ perception. DW at the individual level is interdisciplinary in management and psychology. During the past few years, researchers have been devoted to explaining the definition of DW from the perspective of the Psychology of Working Theory (PWT) [[21], [22], [23]], which fulfills the basic needs of survival, self-determination, and social integration [24]. Unlike the macro perspective, the micro-level of DW emphasizes individual self-value, which focuses on work meaning and work value [25], emphasizing that individuals can realize their self-value and dignity through work challenges and opportunities.

### 2.2 Measurement

#### 2.2.1 Measurement based on macro definition

According to the ILO's definition of DW, four dimensions were identified in the report submitted to the ILO Conference in 1999. Ghai et al. developed four factors of DW in 2003: Employment, social protection, fundamental rights, and social dialogue.

Using the ILO's definition of DW, Ferraro et al. developed the decent work questionnaire (DWQ) for KWs in 2018 [26]. Seven dimensions containing a 31-item scale were developed. They are the basic principles and work values, adequate working time, productive work, fulfillment of labor rights, social protection, health and safety, and opportunities. This instrument measures workers' subjective perceptions of DW conditions in their current jobs at the individual level [27].

#### 2.2.2 Measurement based on micro definition

Based on a macro definition of decent work, Ferraro's scale proposes the microstructure of DW, resulting in research limitations. In order to overcome this research limitation, scholars have developed micro-level decent work scales from the PWT. Conducting the research on a sample of 589 participants, Duffy et al. introduced the decent work scale (DWS) in 2017 [15]. The scale contains five dimensions: working safety, health care accessibility, fair compensation, hours for leisure and rest, and organizational values. The scale is studied on various subjects: technicians, KWs, line workers, and informal workers. Although this scale is credible and valid, it is only developed in the United States. Thus, more samples from other nations and regions in different working contexts are needed to test validity [28]. In recent years, DWS has been validated in Turkey, France, Sub-Saharan Africa, and Australia [[29], [30], [31], [32]].

Based on the PWT and living wage, scholars have conducted in-depth studies at the micro level of DW. They develop DW into five dimensions: reproductive-material, social-communication, legal institutional rights, social status and recognition, and meaningfulness at work [33]. However, it is a conceptual model of DW from the perspective of living wages since the scale has yet to be developed. It is an excellent attempt to understand DW from a living wage perspective based on the PWT.

Duffy and Seubert developed a DWS based on the micro definition in 2017 and 2021. Therefore, both are very important when studying DW at the micro level. Duffy's scale is designed to measure general employees' DW. It needs to consider the differences between knowledge and general workers regarding output diversity, the low independence when filling in the information, and the importance of spatial layout and materials.

Some representative results of the scale developments and dimensions analyzed are shown in Table 1. Above all, researchers developed scales and dimensions based on macro and micro definitions. With the advent of the knowledge economy, knowledge employees are not only characterized by creativity and innovative behavior but also motivated by the job rather than the organization. Consequently, existing scales cannot accurately measure KWs' perceptions of DW, and a DWPS is urgently needed.

**Table 1:** Decent work dimension.

Research level	Researchers	Dimensions
Macro-level	ILO,1999	Employment
		Social protection
		Basic rights
		Social dialogue
	Ferrara et al., 2018	Work values
		Adequate working time
		Productive work
		Fulfillment of labor rights
		Social protection
		Opportunities
		Health and safety
Micro-level	Duffy, 2017	Working safety
		Health care accessibility
		Fair compensation
		Hours for leisure and rest
		Organizational value
	Seubert, 2021	Reproductive material
		Social communication
		Legal institutional rights
		Social status and recognition
		Meaningfulness at work

## 3 Initial scale construction based on grounded theory

Although research on the definition, scale development, and empirical research on DW has been conducted for almost 20 years, the perception of decent work for KWs is an emerging construct in the changing working context. Exploring a DWPS requires a predominantly qualitative research approach. According to Glaser (1967), grounded theory is one of the most effective ways to sprout concepts and theories for a new concept and scale development. This study focuses on coding in-depth interview information using grounded theory to obtain the structural dimensions of knowledge workers' DWP.

### 3.1 Research design

Aiming to extract DWPS items, we conducted in-depth interviews to understand the basic structure of individuals' DWP. As a result, we collected the initial items by following steps: (1) we carried out an in-depth interview with KWs for at least 30 min for each interviewer and recorded their answers to the questions; (2) DWPS was developed based on a review of literature and an analysis of theoretical and empirical research according to DW definition and scale development.

The interview was semi-structured with a specific outline of demographic information: the status quo of DW, the structures of DW, and the effect of DW. The interviewer explained the definition of DW and asked the questions in the outline. In Table 2, the outline is intended to assist the interviewees through the process of thinking, analyzing, and explaining questions that need to be addressed. Due to the semi-structured interview, the interviewers refer to the questions in Table 2. Following the interviewee reactions and responses, the interviewers adjusted the questions.

**Table 2:** Interview questions on DWP.

Theme	Main Content
Demographic information	Gender, age, education, marriage status, yearly income, tenure
Status quo of decent work	Do you think your current job is decent, and why?
Structures of decent work	What aspects have reduced or increased your perception of the job being decent, and why?
Effect of decent work	What kind of work do you think is decent, and why?

### 3.2 Ethics statement

According to the related provisions of the human survey, this study was approved by the Ethics Committee of Shanghai Dianji University. All participants were given oral consent according to the Declaration of Helsinki. In the process of investigation, we told the interviewees that all the oral and written materials would be recorded and kept confidential. All participants were conducted anonymously, and the results will be used for academic research only, not for commercial purposes. We fully respected the wishes of the interviewees.

### 3.3 Procedure

#### 3.3.1 Participants

According to the research requirements, we need KWs as research subjects. In this research, KWs employ and develop concepts, ideas, and theories gained through systematic education rather than someone who employs manual or physical labor to accomplish tasks [34]. We conducted in-depth interviews from May 20th to June 5th, 2022. In-depth interview data collection took 16 days. Before conducting the interview, we learned about their job responsibilities. All the subjects were from professional and management positions, consistent with research purposes. According to the definition of KW, we selected 27 interviewees from banking, securities investment, insurance, communication and information services, real estate, automobile manufacturing, universities, and industries with high levels of knowledge. The interviewees were engaged in management, professional and technical positions requiring knowledge creation and dissemination at work. Participants were recruited voluntarily from the organization that allowed their participation.

Interviewees were randomly selected by gender, industry, income, age, education, tenure, and marriage status. 44% of respondents were female and 56% were male; 34% were between the ages of 25 and 35; 39% were between the ages of 36 and 45; 27% were over 46; and 81% had completed undergraduate studies or higher. Moreover, interviewees had different tenures, incomes, industry backgrounds, and marital statuses. The sample was representative.

#### 3.3.2 Coding procedure

Step 1 is label extraction. As a result of converting the interview recordings into text, we obtained approximately 110,000 words of interview text records. Original codes, an expression of a whole meaning from the intercepted source material, were extracted by translating the original words of the interviewees. Then, the original codes needed to be converted into labels that retain the underlying meaning and be more concise. Using the sentences as a starting point, the researchers compiled phrases and words about decent work, then generated a conceptual model from them. The rule for conversion of original codes to labels was “Which aspects do the interviewees talk about in terms of perceptions of decent work?” Finally, 866 labels were derived from the interview records according to data analysis.

Step 2 is item extraction. Thirty-two items were extracted from 886 original labels. After deleting labels with frequencies less than 3, the researchers discussed each item's expression. They decided to reclassify them according to the definition of DW and KWs' characteristics. Thirty-two items included job stability, income stability, benefits, equity, and professional skills proposed by the ILO, and power, recognition, freedom, autonomy, self-value, opportunities, and achievement closely related to knowledge workers.

Step 3 is dimension extraction. The individual perception of job security is necessary for DWP [35]. Research results show that work security, favorable working conditions, and employment security are the main categories of job security (JS). Work security refers to the physical need to feel protected and safe. Favorable working conditions mean the environment and materials are guaranteed. Employment security refers to the employee not fearing losing their job and benefits psychologically from a job grant [20]. According to the extracted results of dimensions, in terms of work security, “feel safe about the job, income, and benefits” refers to both physical and economic security; “my work materials are secure” and “my working environment is pleasant” refers to a guarantee of working conditions; “feel fair about the distribution system” and “my employer has a good reputation” refers to a sense of psychosocial employment security. We summarized these statements about work security, working conditions, and employment security as “job security,” resulting in 8 scale items.

Respect & support (R&S) is the second dimension of DWP. Recent research suggests that subjective dimensions of DW, such as fairness, challenge, and mastery, can explain the content of DW for blue-collar workers [36]. According to this study, both “feel fair about income and payment” and “have fair treatment at work” refer to the fairness perception. “Feel respected and recognized at work” and “be satisfied with the current job” are related to the perception of respect and recognition of the job, which indicates that the sense of respect and dignity in the workplace is the goal of DW according to previous research [25]. “Have power and resources at work,” “have a positive relationship with colleagues,” and “can get help and support in the workplace” indicate the perception of support in the workplace, which is a positive predictor of practical workability [37]. Perception of fairness, respect, and support were summarized from the original materials as the “respect & support” dimension of the DWP consisting of 8 items.

Self-value (SV) is the third dimension of DWP. The sense of value in the workplace refers to personal and social self-value, such as maximizing one's ability and expanding networks [38]. “My job brings me a good work image and social status” and “I can create value in my work” indicate self-value from the social aspect, meaning that a decent job can enhance individuals' social status and self-image. Alternatively, “have autonomy and freedom,” “can use talents in the job,” and “have a sense of achievement” refer to self-value from an individual perspective, which indicates that DW can contribute to individuals' self-worth. Social and personal self-value perception were extracted from the original materials as the “self-value” dimension of the DWP, consisting of 9 items.

Professional skills & ethics (PS&E) is the final dimension of DWP. KWs' jobs are undoubtedly innovative and challenging due to their specialized expertise and skills. Items related to “my job requires high competence,” “my job requires specialized knowledge and skills,” and “my job is challenging, innovative, and not easily replaced” can be categorized as professional skills. Research results show that KWs' moral attitudes and organizational ethics are distinctive KM features [39]. “My job requires good work ethics” and “I deal with a high level of customers” indicate that decent work for KWs should include work ethics in line with their characteristics.

#### 3.3.3 Coding results

**Fig. 1:**
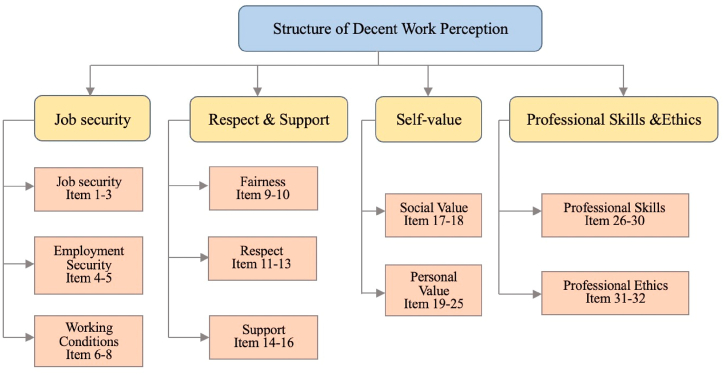
DWP structure.

After reviewing and discussing prior studies, researchers and experts rearranged, categorized, and obtained labels from the original materials to formulate a DWPS comprising 32 items with four dimensions (see Table 3). The first dimension is job security, consisting of 8 items. The respect & support dimension contains 8 items. The third dimension is self-value, and it consists of 9 items. The final dimension is professional skills and ethics, reflecting KWs' characters and comprising 7 items. The structure of DWP is shown in Fig. 1. Qualitative research methods through in-depth interviews are necessary to enhance the theoretical value of DWPS assessment. In the next part, quantitative research methods are conducted to test the credibility and validity of DWPS, enhancing the practical value of this study.

**Table 3:** Classification of items and dimensions.

NO.	Item	Dimensions
1	My job is stable.	Job Security
2	My income is stable.	
3	The job can provide me with good benefits.	
4	I feel fair about the distribution system in my organization.	
5	My employer has a good reputation.	
6	My working materials are secured.	
7	My work is legal.	
8	My working environment is pleasant.	
9	My income and payment are matched.	Respect & support
10	I can enjoy the fair and equitable treatment at work.	
11	I can be respected and am satisfied with my current job.	
12	My work can be valued.	
13	My work allows me to be respected and recognized.	
14	I have power and resources at work.	
15	I can get help and support in my work.	
16	I have a good relationship with my colleagues in the workplace.	
17	My job brings me a good work image.	Self-value
18	My job brings me a high social status.	
19	I can work with autonomy and freedom.	
20	My job can realize my value.	
21	I can create value in my work.	
22	I can use my talents in my work.	
23	My work keeps me healthy and happy.	
24	My work gives me a sense of achievement.	
25	My job gives me ample opportunities for growth and development.	
26	My job requires high competence	Professional Skills and Ethics
27	My job is innovative	
28	My job requires professional knowledge and skills	
29	My job is not easily replaced	
30	My job is challenging	
31	My job requires good work ethics	
32	I deal with a high level of customers

## 4 Quantitative method

### 4.1 Preliminary survey and extraction of DWPS

#### 4.1.1 Participants selection

Preliminary survey is conducted to evaluate the validity of the initial scale and to extract items from the data. The formal DWPS was developed based on preliminary survey results. We conducted a preliminary survey of KW from 10th June to 20th July 2022 in Shanghai, Beijing, Guangzhou, Chengdu, and other developed cities in China. Initially, we published the questionnaire on the website through rolling snowball sampling and invited people to participate. Subjects were encouraged to forward the questionnaire to their colleagues. In the end, we combined the samples and analyzed the sample demographics. Moreover, we provided feedback on research results for the KWs to ensure their active participation in the project.

The essential data in the questionnaires was subjected to a descriptive statistical analysis. 99.9% of the subjects had a bachelor's degree or above, consistent with the study purpose. 212 questionnaires were obtained. We invalidated and deleted questionnaires with incomplete responses or with more than five identical options continuously. As a result, 184 valid questionnaires were collected (86.8%). Descriptive statistical analysis showed 47.3% were males, and 52.7% were females. There were 6.0% of individuals below the age of 25, 49.5% between the ages of 26 and 35, 39.1% between the ages of 36 and 45, and 5.4% over the age of 46. 54.8% of participants had obtained bachelor's degree, 28.8% with master's degree, and 16.3% with doctoral degree. The distribution of industries was mainly concentrated in knowledge-intensive industries, with banking at 16.3%, securities investment at 8.2%, insurance at 23.9%, consulting services at 4.3%, internet technology at 4.9%, real estate at 2.7%, manufacturing at 5.4%, education of 23.9%, and others of 10.3%. Subjects from state-owned enterprise accounted for 42.4%, private enterprise accounted for 20.1%, foreign-funded enterprise accounted for 12.5%, public institutions were 23.9%, and governments were 1.1%. 9.2% of the sample were lower than a half year of tenure, 5.4% were 0.5–1 year, 20.1% were 1.01–3 years, 11.4% were 3.01–5 years, 19.6% were 5.01–10years, more than 10.01 years was 34.2%.

#### 4.1.2 Procedure

The first step is to conduct a reliability assessment on the original scale. (1) CICT is used as a standard for reliability tests. The items should be removed when CICT is less than 0.3. Items 1 and 7 from JS were deleted because CICT was smaller than 0.3. If items with CICT are between 0.3 and 0.5, Cronbach'α of the scale increases when items are deleted. That means these items should be deleted. Items 9 and 16 from the R&S dimension and 31 and 32 from the PS&E dimension were deleted because the CICT was between 0.3 and 0.5. The Cronbach'α raised when these two items were deleted. Consequently, 26 items were retained since the CICT of six items was not qualified (see Table 4). (2) Descriptive statistical analysis. We conducted descriptive analysis on initial data to test the quality of each item. The standard deviation of any item is greater than 0.75, indicating no low-discrimination problem. (3) Extreme group test. In a preliminary survey of 212 KWs, we chose 27% of the candidates with the highest values and 27% of the candidates with the lowest values. A *t*-test was carried out for the two groups. Based upon the *t*-test results, all items were able to effectively distinguish. So, all the items were retained. (4) Correlation test. Each of the 26 items are significantly correlated with the initial scale. (5) Cronbach's α test. The results show that Cronbach's α are greater than 0.7 after deleting items. Therefore, the DWPS consists of 26 items.

**Table 4:** Reliability test for initial DWPS.

Dimension	Item	CITC	Cronbach'α after deleting item	Cronbach'α	Dimension	Item	CITC	Cronbach'α after deleting item	Cronbach'α
Job Security	JS1	0.292	0.839	0.826	Self-Value	SV1	0.674	0.914	0.902
	JS2	0.597	0.801			SV2	0.569	0.920	
	JS3	0.684	0.786			SV3	0.629	0.918	
	JS4	0.615	0.796			SV4	0.821	0.904	
	JS5	0.672	0.789			SV5	0.725	0.911	
	JS6	0.624	0.796			SV6	0.696	0.912	
	JS7	0.372	0.826			SV7	0.699	0.912	
	JS8	0.561	0.805			SV8	0.865	0.901	
Respect & Support	R&S1	0.485	0.886	0.882		SV9	0.777	0.907	
	R&S2	0.724	0.859		Professional Skills & Ethics	PS&E1	0.579	0.819	0.839
	R&S3	0.430	0.885			PS&E 2	0.688	0.801	
	R&S4	0.550	0.877			PS&E 3	0.673	0.804	
	R&S5	0.783	0.852			PS&E 4	0.459	0.845	
	R&S6	0.794	0.851			PS&E 5	0.766	0.786	
	R&S7	0.739	0.857			PS&E 6	0.611	0.815	
	R&S8	0.694	0.862			PS&E 7	0.376	0.850	

The second step is to conduct an EFA. The p-value of the KMO and Bartlett test was 0.000, indicating that 26-item DWPS was adequate for EFA. Then, we conducted a principal component analysis (PCA) on 26 items. We deleted 14 items with loading values lower than 0.4 or cross-loading values greater than 0.4. Based on the results of multiple analysis, items 4, 5, and 8 from JS were deleted; items 11 and 14 from R&S were deleted; items 17, 18, 20, 22, 23, and 25 from SV were deleted, and items 27 and 29 from PS&E were deleted. Consequently, we developed DWPS with 13 items, consisting of 3 items in the JS dimension, 4 in the R&S dimension, 3 in the SV dimension, and 3 in the PS&E dimension. We noted that 3 items from PS&E were about professional skills. Thus, we revised the final dimension as professional skills (PS) on the formal scale.

Finally, We modified the language of the questionnaire in response to participants' feedback and experts' comments. This enhanced the scale expression quality and improved content validity.

To summarize, we significantly enhanced the initial scale quality by using reliability assessment and EFA. Finally, we obtained a 13-item DWPS used as a formal scale (see Appendix).

### 4.2 Formal survey and structural analysis of DWPS

#### 4.2.1 Data collection

We collected data by questionnaire from 1st September to 30th November 2022. The formal survey took three months. 585 formal questionnaires were sent out, 554 were effective, and the efficacy of recovery was 96.4%. We conducted descriptive statistics of the formal sample. The results indicated that 87.4% of subjects had bachelor's degrees or above, which met study requirement. The specific sample distribution is shown in Table 5.

**Table 5:** Descriptive analysis of formal sample.

Gender	Frequency	Industry	Frequency
Male	41.2	Banking	22.4
Female	58.8	Insurance	42.4
Age	Frequency	Securities investment	4.2
<25	7	Telecommunication and internet service	6.0
26–35	57.2	Education	21.7
36–45	26.9	Others	3.4
>46	8.9	Ownership	Frequency
Tenure	Frequency	Private enterprise	8.1
<0.5 year	6.0	State-owned enterprise	46.8
0.5–1 year	5.6	Public institutions and governments	26.4
1.01–3 years	23.6	Foreign-funded enterprise	8.1
3.01–5 years	19.0	Others	10.6
5.01–10 years	19.1		
>10.01 years	26.7		

#### 4.2.2 Exploratory factor analysis

We carried out an EFA on half of the sample (N = 277) by software SPSS 26.0. According to the results, KMO test result was 0.941 and the p-value of Bartlett test was significant, suggesting that the variables were significantly associated and adequate for factor analysis. We developed the factor loading matrix by conducting PCA as shown in Table 6. The 4-factor model accumulated variance explanation rate was 81.851%, indicating that the extracted four factors could explain 81.851% of the information content of the total 13 items. The explained variance of the four factors was 21.969%, 21.443%, 19.694%, and 18.743%, respectively.

**Table 6:** EFA results.

Item	Factor			
	S1	S2	S3	S4
JS1	0.803			
JS2	0.827			
JS3	0.782			
R&S1				0.491
R&S2				0.654
R&S3				0.591
R&S4				0.874
SV1			0.609	
SV2			0.714	
SV3			0.661	
PS1		0.769		
PS2		0.864		
PS3		0.806		
Factor variance contribution %	21.969	21.443	19.694	18.743
Accumulated variance contribution %	21.969	43.413	63.107	81.851

After combining the items and analyzing the literature, we defined the four dimensions of DWPS as follows:

(1) “JS” (3 items) is the overall perception of KWs about income stability, workplace security, and benefits.
(2) “R&S" (4 items) is a holistic perception of KWs about fairness, respect, support, and recognition at work.
(3) “SV” (3 items), as the KW's sense of autonomy at work, value creation, and achievement at work.
(4) “PS” (3 items), as the individual's perception of challenging and professional skills application at work.

#### 4.2.3 Confirmatory factor analysis

We used the second half of the sample (N = 277) to test whether the conceptual model of DWPS fitted the actual observed data or not. Four competition models were proposed below to better verify the model's accuracy. They were:

M1: Single-factor model in which we proposed that 13 items shared a joint latent variable (LV): DWPS.
M2: We hypothesized that JS and R&S would share the same LV, and SV and PS would have the same LV.
M3: We hypothesized that JS would have one LV, R&S would have one LV, and SV and PS would share the similar LV.
M4: 4-factor model was consistent with EFA. We proposed that the 4-factor DWPS, JS, R&S, SV and PS, would have a significant impact on this model.

**Fig. 2:**
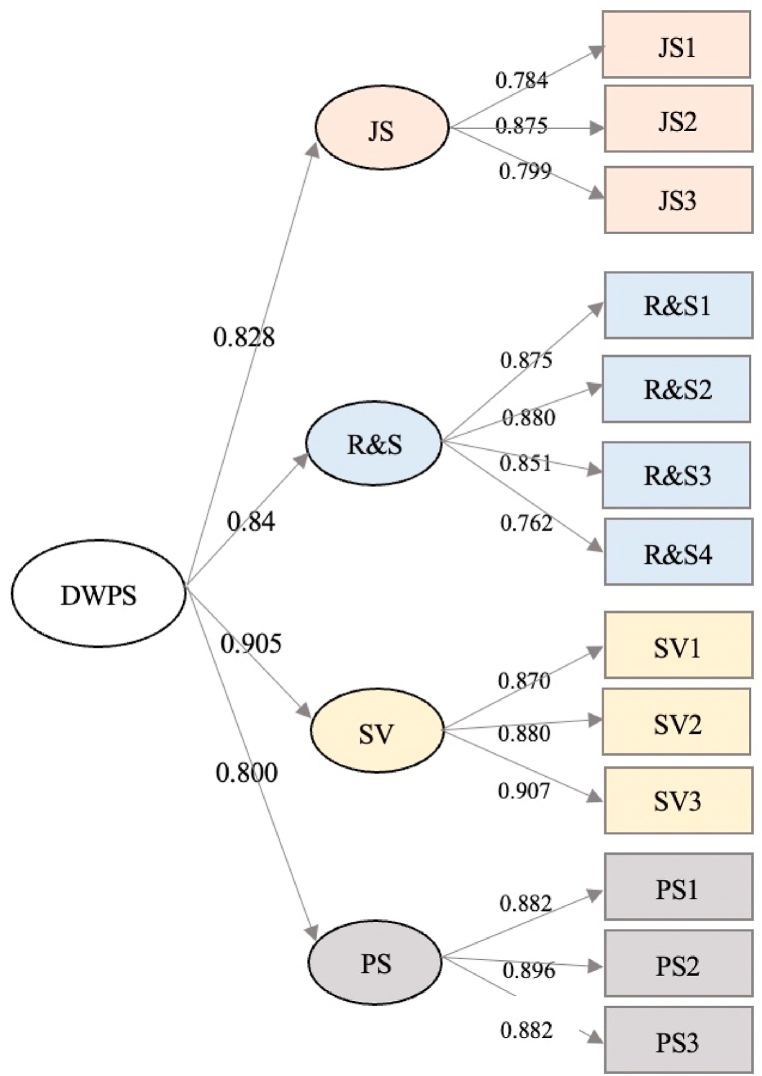
Path coefficient of CFA.

Each factor served as a LV and related items served as an observational variable among the four models. We conducted a CFA to test the model fit (MI). Table 7 shows MI values for M1, M2, and M3 that are unacceptable. GFI, CFI, and NFI were less than 0.9, and the RMSEAs of the three models were greater than 0.1. According to the MI value of M4, χ2/df of 3.929 was the smallest among the four models, which was ideal for MI. GFI, CFI, and NFI were more significant than 0.9, and the RMSEA of the M4 model was 0.1. Thus, we consider M4 as the optimal model (see Table 7), and the path coefficient of confirmatory factor analysis is shown in Fig. 2.

**Table 7:** Major fitting degree indices of DWP.

Value	χ2	df	χ2/df	GFI	RMSEA	RMR	CFI	NFI
M1: Single-factor model	855.113	65	13.156	0.602	0.210	0.063	0.739	0.725
M2: 2-factor model	476.2	64	7.441	0.752	0.153	0.042	0.864	0.847
M3: 3-factor model	373.554	62	6.025	0.802	0.135	0.038	0.897	0.880
M4: 1-factor model	239.689	61	3.929	0.878	0.100	0.041	0.941	0.923

#### 4.2.4 Reliability test and validity test

Reliability can be evaluated from two aspects: the scale credibility and LV credibility. In general, Cronbach's α is used to test scale credibility, and CR value is used to test LVs' credibility. DWPS's reliability value was 0.945(N = 554), which was greater than 0.7, suggesting that it was credible. Moreover, Cronbach's α values for each LV were 0.869, 0.902, 0.912, and 0.902, respectively, suggesting that all LVs were highly credible. A total of 0.860, 0.908, 0.916, and 0.917 CR values were found for each LV, indicating that all LVs passed the reliability test. DWPS had reasonable reliability because each principal component was not taken as a single variable and had limited items.

Validity tests are conducted on content validity and structure validity. By evaluating DWPS content validity, qualitative methods ensure its appropriateness and conformity. We strictly adhered to the questionnaire development rules and process. We conducted an extensive literature collection and review. We collected data through in-depth interviews, invited managers and scholars from HRM to discuss each item's expression in the questionnaire, and conducted a preliminary survey utilizing 212 questionnaires. Therefore, the content validity of the study was reliable.

The structural validity test is another part of the validity test. Verifying structural validity involves examining scale convergence and discriminant validity. The standardized load of 13 items was more significant than 0.7, indicating that each factor was closely related to its items. Meanwhile, AVE of each factor was larger than 0.6, suggesting good convergence validity (see Table 8). The square root of AVE is greater than the correlation coefficient for every LV (see Table 9), indicating that DWPS had significant discriminant validity.

**Table 8:** Convergence validity test result.

Factor	Item	Std. Estimate	CR	AVE
JS	JS1	0.784	0.860	0.673
	JS2	0.875		
	JS3	0.799		
R&S	R&S1	0.875	0.908	0.711
	R&S2	0.880		
	R&S3	0.851		
	R&S4	0.762		
SV	SV1	0.870	0.916	0.785
	SV2	0.880		
	SV3	0.907		
PS	PS1	0.896	0.917	0.786
	PS2	0.882		
	PS3	0.882

**Table 9:** Discrimination validity test result.

	JS	RS	SV	PS
JS	0.820*			
RS	0.720	0.843*		
SV	0.611	0.695	0.886*	
PS	0.557	0.532	0.732	0.887*

## 5 Conclusion and discussion

### 5.1 Conclusion

(1) We reviewed previous literature on DW and interviewed 27 KWs by in-depth interview. Through qualitative analysis, we conducted the initial 32-item scale of DWPS. Afterward, we obtained the items by coding and verified the DWPS dimensions based on PCA. Then we conducted a formal survey and obtained a 13-item DWPS.
(2) 554 formal survey questionnaires were collected. JS, R&S, SV and PS were obtained from EFA of half of the data (N = 227). KMO was 0.941 and had 81.851% cumulative variance of the four factors, indicating that the four factors adequately explained DW. Second half of the 227 questionnaires were used for CFA. The four-factor model proved the most effective of the four models. Meanwhile, the four-factor model's GFI, CFI, and NFI values were 0.878, 0.941, and 0.923, respectively. The RMSEA was 0.1 and χ2/df was 3.929, indicating that the model was well fitted. In summary, EFA and CFA results showed that DWPS had a reasonable fit.
(3) The reliability and validity tests were carried out on a formal survey. Cronbach's α of the overall credibility of DWPS was 0.945, more significant than 0.7, indicating that the scale was reliable. Therefore, Cronbach's α of each LV was 0.869, 0.902, 0.912, and 0.902 respectively. The CR value was 0.860, 0.908, 0.916, and 0.917, respectively. We strictly adhered to the scale development procedure and invited professors and experts from HRM to review and revise the item expressions. The scale development procedure was scientific and rigorous. Content validity could be reliable. The standardized loading value of 13 items was more significant than 0.7, indicating good convergence validity. The root of the AVE value was 0.82, 0.843, 0.886, 0.887 respectively. Each of them is greater than the correlation coefficient between variables, showing high discrimination validity.

### 5.2 Theoretical implication

This paper developed a four-dimensional DWPS for KWs: JS, R&S, SV and PS. Scholars have developed measurement tools to perceive DW in the past 20 years [23,26]. Ferraro et al. (2018) developed a 7-dimensional model of DW for knowledge workers and contributed to measuring DW [26]. The scale development was conducted on the macro-level concept of DW from the ILO, focusing on basic rights and social justice. However, the research subjects were KWs who were specialized in innovative behavior and motivated by their job at the micro-level. The gap between DW macro concept and micro subjects reduced the scale applicability. Based on PWT, Duffy et al. (2017) developed a micro-level scale for mass workers consistent with the micro definition of DW [23]. However, the scale developed by Duffy et al. (2017) was unsuitable for KWs because they ignored professionalism and meaningfulness. Based on the micro-level definition of DW, this study develops DWPS for KWs by considering innovative behavior and professional commitment features. This study extends the scope of DW research by linking DW to knowledge management and contributing to the new understanding of DW structures.

First, in workplace security, this paper constructs and tests JS as the first dimension of DWP, including 3 items: income security, work safety, and benefits security. This conclusion is consistent with previous research results [23]. Although the concept of DW originated from the past 20 years, this concept is facing a huge challenge in modern times posed by underemployment, wage security, and atomization at work [16]. JS is the fundamental element of DW in various groups across different nations and countries.

Second, in workplace psychology, this study constructs and tests R&S and SV associated with DWP, reflecting challenges and autonomy at work. According to the 4-itme R&S dimension, KW values self-respect and support from their employers and fair treatment. SV dimension with 3 items includes work autonomy, value creation, and sense of achievement. These elements reflect the psychological characteristics of KW, including their difficulty monitoring their work processes and the high level of autonomy they enjoy at work. Accordingly, this study's new findings are fair treatment from the R&S dimension, work autonomy, value creation, and a sense of accomplishment from the SV dimension. Fair treatment from the R&S dimension is consistent with difficulty monitoring work processes. Knowledge creation encounters many difficulties and requires much time and resources to solve problems. Fair treatment reflects respect for the involvement of KW. SV, reflecting high autonomy at work, is an emerging dimension of decent work perception. High work autonomy builds a free and relaxed working atmosphere, facilitating knowledge creation and self-determination.

Lastly, regarding specialized skills in the workplace, this study constructs and tests 3 items within the PS dimension of the DWP, reflecting the career commitment of KW. PS can be measured by the application of specialized knowledge, the response to job requirements in terms of professionalism, and work challenges. This dimension is derived from the ability of KW to manage and enhance their knowledge and expertise in the workplace and to use this ability to proactively solve work challenges, thus emphasizing that KWs are critical organizational assets. Therefore, high job requirements and work challenges are new elements of DWP, reflecting career commitment characteristics from the perspective of specialized skills. Skills based on human capital could be enhanced, and resources based on career capital can be accumulated through meeting high job requirements and completing work challenges. As a result, KWs have a strong sense of achievement and become more committed to their careers.

### 5.3 Practical implication

HRM practices in China have been facing problems of professional skills and management systems, which affects the management practices efficacy. Based on this realistic demand, this study attempts to provide a scientific scale for measuring DW for managers and government officials. It also offers a pathway to increase the awareness of social justice on the work-life quality and well-being of KWs, on the other hand.

To begin with, we offer a practical and scientific scale to measure DW for KW. Managers may find this scale beneficial in understanding KWs' needs When they address work-life well-being. Using DWPS, government officials may determine whether KW's jobs meet the ILO's standard. Additionally, managers and government officials can assess KWs' access to the specific DW dimension and tailor their interventions based on the assessment results. While DWPS is expected to be an effective measurement tool in management practices, focusing on KWs' perception of DW can help improve their work meaningfulness and their sense of well-being at work.

Alternatively, DWPS offers a pathway to increase the awareness of social justice on the work-life quality and well-being of KWs. We propose that an organic integration of KWs’ features into DWP is warranted. KWs change from pursuing job security to meaningful work [40]. As a result of this subtle change, KWs are transferring their behavior patterns, enabling self-respect, achieving work meaningfulness, and realizing their career goals. Therefore, DWPS may be used as a tool to engage KWs in the discussion around work life quality. Thus, DWPS can be seen as an effective tool to influence social justice. For instance, a knowledge worker who considers the working condition as unsatisfied can use this measurement tool to find out the potential contextual factors (e.g., overwork, low self-value) affecting his or her feeling about work and empower him or her to improve work conditions.

## 6 Limitations and future research directions

The limitations are illustrated as below. (1) Sample distribution limitations. Although this study selects samples from developed cities in China, considering that economically developed areas tend to gather more samples, some developed areas are not included. (2) Limitations of empirical tests. DWPS for KWs has been developed in this study, but not verified by empirical studies. Therefore, the scale must be further examined, modified, and optimized in future research.

Due to time and financial constraints, the scale's validity is tested only in Chinese working circumstances. We would like to apply this scale to other working groups and compare KWs from various cultures worldwide in the future due to comparative studies between China and other countries are important [41]. We want to conduct extensive research on DWPS in various countries and regions. In future research, it is necessary to test the applicability of measurement scales across multiple industries, different types of organizations, and different types of careers.

## Author contribution statement

Y.Y: Conceived and designed the analysis; Wrote the paper; Y.G: Analyzed and interpreted the data; Contributed analysis tools or data; G.J: Contributed analysis tools or data; Wrote the paper.

## Funding statement

This research was funded by the Annual Project of Shanghai Dianji University for Project Teaching (A1-5101-22-003-08-153).

## Data availability statement

Data will be made available on request.

## Additional information

No additional information is available for this paper.

## Declaration of competing interest

The authors declare that they have no known competing financial interests or personal relationships that could have appeared to influence the work reported in this paper.

## Figures and Tables

**Table:** 

Formal Questionnaire of DWP for Knowledge Workers
My income is secured
My organization can provide security for my work
The organization can provide me with good benefits
I am fairly and equitably treated at work
I can be respected and am satisfied with my current job
I can get help and support in my work
My work allows me to be respected and recognized
I can work with autonomy and freedom
I can create value in my work
My work gives me a sense of achievement
My job requires high competence
My job requires professional knowledge and skills
My job is challenging
