# Corrigendum to “Measuring the decent work of knowledge workers: Constructing and validating a new scale” [Heliyon Volume 9, Issue 7, July 2023, Article e17945]

**DOI:** 10.1016/j.heliyon.2025.e43327

**Published:** 2025-04-14

**Authors:** Yan Yan, Yuqing Geng, Juan Gao

**Affiliations:** School of Business, Shanghai Dianji University, Shanghai, China

In this article, the authors omitted the ethical approval number and date that approval was obtained from the ethical statement in this publication, which is required under the policies of the journal. The authors confirm that ethical approval was obtained from the Ethics Committee of Shanghai Dianji University, for the research under the following reference number: 2022031201, dated 12^th^ March 2022.

The original ethics statement in the article can be found below:

***3.2. Ethics statement***

*According to the related provisions of the human survey, this study was approved by the Ethics Committee of Shanghai Dianji University. All participants were given oral consent according to the Declaration of Helsinki. In the process of investigation, we told the interviewees that all the oral and written materials would be recorded and kept confidential. All participants were conducted anonymously, and the results will be used for academic research only, not for commercial purposes. We fully respected the wishes of the interviewees*.

The statement has been updated to include the ethical approval reference number and date.

The correct version of the ethics statement should be as below:

***3.2. Ethics statement***

*According to the related provisions of the human survey, this study was****reviewed and****approved by the Ethics Committee of Shanghai Dianji University****with the approval reference number: 2022031201, dated 12*^*th*^*March 2022****. All participants were given oral consent according to the Declaration of Helsinki. In the process of investigation, we told the interviewees that all the oral and written materials would be recorded and kept confidential. All participants were conducted anonymously, and the results will be used for academic research only, not for commercial purposes. We fully respected the wishes of the interviewees*.

The ***authors*** apologize for the errors.

## Declaration of competing interest

The authors declare that they have no known competing financial interests or personal relationships that could have appeared to influence the work reported in this paper.

